# Lithium Toxicity in Two Coronavirus Disease 2019 (COVID-19) Patients

**DOI:** 10.7759/cureus.8384

**Published:** 2020-05-31

**Authors:** Kulachanya Suwanwongse, Nehad Shabarek

**Affiliations:** 1 Internal Medicine, Lincoln Medical Center, New York City, USA

**Keywords:** covid, corona virus disease, lithium, toxicity, case report, kidney failure, aki

## Abstract

Coronavirus disease 2019 (COVID-19) is a highly contagious disease, which is currently causing a devastating pandemic resulting in more than millions of infected cases worldwide. Emerging evidence reports the impact of several co-morbidities on the clinical features and outcomes of COVID-19. However, the evidence regarding the association of mental health illnesses and psychiatric treatment on the prognoses of COVID-19 is still lacking. Lithium is a commonly prescribed psychiatric medication that is also well known for its highly lethal toxicity. Many factors can fluctuate the level of lithium, such as drug interaction, illness, and infection. Prompt recognition and management of lithium intoxication is required to reduce patients’ morbidity and mortality. Currently, there is no report regarding COVID-19 and lithium toxicity. Herein, we are presenting two patients with COVID-19 who initially presented with signs and symptoms of lithium toxicity. Our cases emphasize the need for special attention in taking care of patients who are taking lithium during the COVID-19 pandemic. In general, we recommend obtaining lithium levels in all patients who have been taking lithium and have the diagnosis of COVID-19.

## Introduction

Severe acute respiratory syndrome coronavirus 2 (SARS-CoV-2) infection resulting in coronavirus disease 2019 (COVID-19) is currently a rapidly evolved public health issue, with almost five million people diagnosed and more than 300,000 deaths recorded [1]. Several co-morbidities have been identified as negative prognostic outcomes in patients with COVID-19 [2]. However, there is a lack of evidence regarding the impact of mental health illnesses and psychiatric treatment on the severity and mortality related to COVID-19.

Lithium is a commonly prescribed psychiatric medication in both hospitalized and non-hospitalized patients. Lithium has several clinical benefits and is considering a drug of choice for patients with bipolar disorder [3]. Despite its high effectiveness, lithium has a very narrow therapeutic window and its toxicity can be lethal especially if the toxicity is not recognized. Also, the level of lithium can be affected by various causes, including the interaction with other medications, patients’ illness, and infection. Lithium intoxication leads to numerous fatal complications, such as cardiac arrhythmias, neurological disturbances, endocrine abnormalities, and kidney failure [3,4]. Prompt recognition and management of lithium intoxication will help to limit patients’ morbidity and mortality. To date, there is no report about lithium toxicity in patients with COVID-19. Herein, we are reporting the clinical features and outcomes of two patients with COVID-19 who initially presented with signs and symptoms of lithium toxicity.

## Case presentation

Case 1

A 67-year-old woman with a past medical history of schizoaffective disorder, diabetes mellitus, hypertension, and hyperlipidemia was brought to the mental health clinic by her daughter due to behavior changes. Her medications included lithium 600 mg daily, quetiapine 400 mg twice daily, insulin, empagliflozin, metformin, sitagliptin, atorvastatin, irbesartan, and aspirin. On examination, she was poorly groomed without any acute distress. Her speech was unintelligible. She was disoriented to time, place, and person. Her vital signs were as follows: respiratory rate (RR) 18 breaths per minute (breaths/min) , temperature (T) 98.8 degrees Fahrenheit (°F), heart rate (HR) 100 beats per minute (bpm), blood pressure (BP) 145/82 mmHg, and oxygen saturation (SpO_2_) 93% on room air. Her lung exam revealed mild crepitation. 

**Figure 1:**
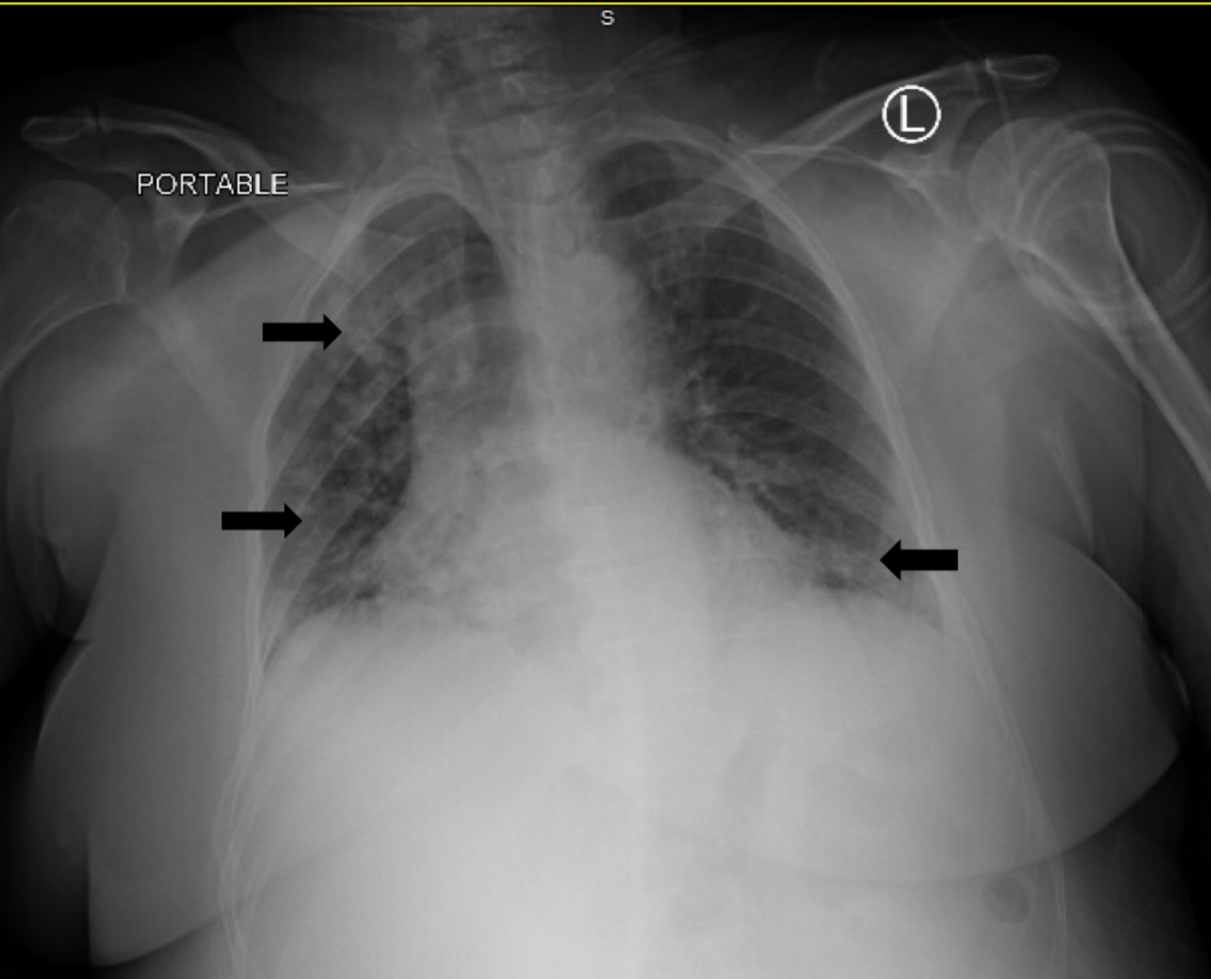
Chest X-ray reveals bilateral infiltration

Laboratory tests showed white blood cell count (WBC) 7.78 x 10^3^ cells per cubic millimeter (/mm^3^) with lymphocytes 9.4%, elevated creatinine (Cr) 1.35 milligrams per deciliter (mg/dl) (baseline 0.65 mg/dl), and lithium level 2.28 mmol/L. Electrocardiogram (ECG) was normal. Computed tomography (CT) of the head was unremarkable. Chest X-ray (CXR) showed bilateral patchy infiltrates as demonstrated in Figure 1. A nasopharyngeal swab for the SARS-CoV-2 polymerase chain reaction test was done and returned positive.

She was admitted to the medicine floor due to COVID-19 pneumonia, acute kidney injury (AKI), and lithium toxicity. On the first day of admission, she received a total of four liters of intravenous normal saline for the treatment of lithium intoxication and AKI. She did not develop acute worsening of COVID-19 symptoms from the administration of intravenous fluid and had good urine output. Table 1 demonstrates the changes in her WBC, Cr, and lithium level during the hospital course. Her mental status was improved, and lithium level was normalized. She received hydroxychloroquine for the treatment of COVID-19 as per hospital protocol; however, her hospital course was complicated with acute hypoxic respiratory failure and eventually died on day 4 of admission. 

**Table 1:** The changes in patient's WBC, % lymphocytes, Cr, and lithium level WBC, white blood cell count; Cr, creatinine

	Day 1	Day 2	Day 3	Day 4
WBC (%L)	9,950 (12)	14,800 (9)	7,780 (9)	10,880 (9)
Cr	1.35	1.94	1.60	1.40
Lithium	2.28	1.8	1.5	1.2

Case 2

An 18-year-old man with a past medical history of bipolar disorder, autistic spectrum disorder, attention deficit hyperactivity disorder, hypothyroidism, and mild persistent asthma was brought to the emergency department by her mother due to alteration of consciousness. His mother also reported that he had a fever, nasal congestion, and cough for seven days. His current medication included lithium 450 mg twice daily, clozapine 100 mg daily, and levothyroxine. On examination, he was somnolence but arousable. He was not in acute distress and could answer simple questions and oriented. His vital signs were as follows: RR 18 breaths/min, T 101.3°F, HR 120 bpm, BP 120/60 mmHg, and SpO2 98% on room air. His lung exam was normal.

**Figure 2:**
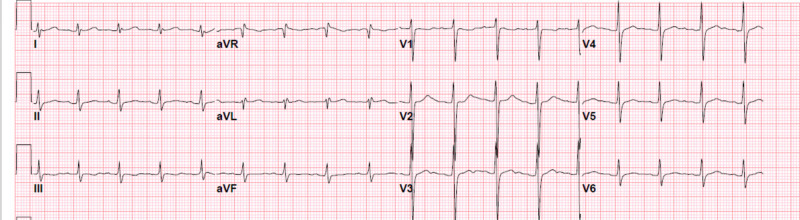
Electrocardiogram shows sinus tachycardia

Laboratory tests showed WBC 11.79 x 10^3^/mm^3^ with lymphocytes 17%, elevated Cr 1.81 mg/dl (baseline 1 mg/dl), and lithium level 2.60 mmol/L. ECG showed sinus tachycardia with an HR of 107 bpm, as demonstrated in Figure 2. CXR was normal. A nasopharyngeal swab for the SARS-CoV-2 polymerase chain reaction test returned positive.

He was admitted to the pediatric intensive care unit due to COVID-19 infection, AKI, and lithium toxicity. Aggressive intravenous normal saline was administered for the treatment of lithium intoxication and AKI. He did not receive hemodialysis. His mental status was improved. Table 2 illustrates the dynamic change in his WBC, Cr, and lithium level. Cr and lithium levels were normalized. He received symptomatic treatment for the COVID-19 infection. His conditions were resolving, and he was discharged home without any complications on hospital day 11. He did not develop hypoxemia throughout the hospital course. Lithium was discontinued with a plan to resume on the follow-up visit. 

**Table 2:** The changes in patient's WBC, % lymphocytes, Cr, and lithium level WBC, white blood cell count; Cr, creatinine

	Day 1	Day 2	Day 3	Day 4
WBC (%L)	11,790 (8)	Not done	Not done	8,700 (24)
Cr	1.66	1.02	1.05	0.95
Lithium	2.6	1.9	1.0	0.6

## Discussion

Lithium is a highly water-soluble ion, well absorbed by the gastrointestinal tract and excreted mostly by the kidneys [5]. Thus, the clearance of lithium largely depends on renal function and volume status. Lithium is well recognized for its potency as well as its narrow therapeutic index. Therapeutic ranges of lithium are between 0.8 and 1.2 mEq/L, while the levels of 1.5 and above are associated with its toxicity [5]. Mental status changes and neurological symptoms are the most common presentations of lithium intoxication in the emergency department [4,5]. 

We report two patients initially presented with acute lithium intoxication in the setting of SARS-CoV-2 infection. To date, there is no evidence support or against the relationship between lithium toxicity and COVID-19. Our patients had clinical features consistent with lithium toxicity including confusion, behavior change, and changes in mental status. Both patients’ mental status was improved after receiving aggressive intravenous fluid and did not require hemodialysis. However, the first patient had later developed severe COVID-19 and passed away from acute hypoxemic respiratory failure. The second patient had mild COVID-19 infection but complicated with AKI and lithium intoxication. Prompt diagnosis and treatment of lithium intoxication in the second patient has led to a favorable prognosis. 

Our report may point out that the clinical characteristics and outcomes of COVID-19 in patients with psychiatric illness and taking lithium are similar to those of the general population. Patient 1 was an older adult with several co-morbidities proven to increase risks of COVID-19 mortality and had significant changes in her CXR. She later developed severe COVID-19 and death. In contrast, patient 2 is a teenager and had normal CXR. He had mild COVID-19 and later resolved without complications. More research elucidates the relationship between lithium toxicity and COVID-19 will be worthwhile. 

Our cases also highlight the need for special attention in taking care of patients who are on lithium or any medications with a narrow therapeutic index during the COVID-19 pandemic. Patients with COVID-19 are likely to have poor oral intake, dehydration, and declined renal function. Some patients may be quarantined at home due to mild symptoms of COVID-19 but at risk of lethal complications from medication toxicity. Clinicians should be aware of the possibility of drug toxicity in suspected COVID-19 patients, especially patients who are on medication with narrow therapeutic ranges. Drug levels should be obtained in COVID-19 patients who have a high risk for medication toxicity, such as patients with multiple co-morbidities, elderly, and polypharmacy. Prompt recognition and treatment of medication toxicity will prevent patients' morbidity and mortality. If possible, we would recommend obtaining lithium level in every suspected COVID-19 cases regardless of severity. As these patients are at risk of developing fatal complications from lithium intoxication, early detection and prompt treatment is required to prevent death. 

## Conclusions

Patients with psychiatric illness and taking lithium may have similar clinical features and outcomes of COVID-19 compared to those without. Lithium intoxication is common in patients with acute illness. More research is needed to determine the association between lithium toxicity and COVID-19 infection. Lithium levels should be obtained in all COVID-19 patients who currently taking lithium, particularly those of high risks. Rapid recognition and management of lithium intoxication is required to limit patients’ morbidity and mortality. 
